# Genistein Supplementation and Cardiac Function in Postmenopausal Women with Metabolic Syndrome: Results from a Pilot Strain-Echo Study

**DOI:** 10.3390/nu9060584

**Published:** 2017-06-07

**Authors:** Cesare de Gregorio, Herbert Marini, Angela Alibrandi, Antonino Di Benedetto, Alessandra Bitto, Elena Bianca Adamo, Domenica Altavilla, Concetta Irace, Giacoma Di Vieste, Diego Pancaldo, Roberta Granese, Marco Atteritano, Salvatore Corrao, Giuseppe Licata, Francesco Squadrito, Vincenzo Arcoraci

**Affiliations:** 1 Department of Clinical and Experimental Medicine, University of Messina, 98100 Messina, Italy; cdegregorio@unime.it (C.d.G.); hrmarini@unime.it (H.M.); adibenedetto@unime.it (A.D.B.); abitto@unime.it (A.B.); jackydv@hotmail.it (G.D.V.); matteritano@unime.it (M.A.); fsquadrito@unime.it (F.S.); 2 Department of Economics, Business, Environmental Science and Quantitative Methodologies, University of Messina, 98100 Messina, Italy; aalibrandi@unime.it; 3 Department of Experimental, Specialized Medical and Surgical and Odonto-stomatological Sciences, University of Messina, 98100 Messina, Italy; elenabianca.adamo@unime.it; 4 Department of Pediatric, Gynecological, Microbiological, and Biomedical Sciences, University of Messina, 98100 Messina, Italy; daltavilla@unime.it (D.A.); roberta.granese@unime.it (R.G.); 5 Department of Clinical and Experimental Medicine, University Magna Græcia, 88100 Catanzaro, Italy; irace@unicz.it; 6 Department of Cardiology, SS. Annunziata Hospital, 12038 Savigliano (CN), Italy; pancaldodiego@libero.it; 7 Centre of Research for Effectiveness and Appropriateness in Medicine (C.R.E.A.M.), Di.Bi.M.I.S., University of Palermo, 90127 Palermo, Italy; s.corrao@tiscali.it (S.C.); licatag@unipa.it (G.L.); 8 Department of Internal Medicine, National Relevance and High Specialization Hospital Trust ARNAS Civico, Di Cristina, Benfratelli, 90127 Palermo, Italy

**Keywords:** genistein, metabolic syndrome, menopause, cardiac function, echocardiography

## Abstract

Genistein, a soy-derived isoflavone, may improve cardiovascular risk profile in postmenopausal women with metabolic syndrome (MetS), but few literature data on its cardiac effects in humans are available. The aim of this sub-study of a randomized double-blind case-control study was to analyze the effect on cardiac function of one-year genistein dietary supplementation in 22 post-menopausal patients with MetS. Participants received 54 mg/day of genistein (*n* = 11) or placebo (*n* = 11) in combination with a Mediterranean-style diet and regular exercise. Left ventricular (LV) systolic function was assessed as the primary endpoint, according to conventional and strain-echocardiography measurements. Also, left atrial (LA) morphofunctional indices were investigated at baseline and at the final visit. Results were expressed as median with interquartile range (IQ). A significant improvement of LV ejection fraction (20.3 (IQ 12.5) vs. −1.67 (IQ 24.8); *p* = 0.040)), and LA area fractional change (11.1 (IQ 22.6) vs. 2.8 (9.5); *p* = 0.034)) were observed in genistein patients compared to the controls, following 12 months of treatment. In addition, body surface area indexed LA systolic volume and peak LA longitudinal strain significantly changed from basal to the end of the study in genistein-treated patients. One-year supplementation with 54 mg/day of pure genistein improved both LV ejection fraction and LA remodeling and function in postmenopausal women with MetS.

## 1. Introduction

Estrogen favorably influences calcium homeostasis, serum lipid levels, blood pressure control, inflammatory status, and vascular reactivity in women [1,2,3,4,5]. Data from Italian and other European registries indicate that postmenopausal women presenting with traits of metabolic syndrome (MetS), mostly diabetes mellitus (DM) and hyperlipemia, are at high risk of cardiovascular (CV) events [1,6,7]. Moreover, the use of lipid-lowering therapy is strongly influenced by reimbursement criteria revision, and the female gender was identified as a patient-related predictor of low adherence [8,9,10]. The advantages of estrogen supplementation are preserved in early menopause, failing to maintain cardiovascular protection, thus further therapeutic options continue to be studied [11,12,13]. Genistein aglycone (hereinafter referred to as genistein) is a soy isoflavone, which has progressively gained clinical consideration for the management of postmenopausal symptoms. Its molecular structure resembles that of 17β–estradiol and binds the same estrogen-receptors in a dose-dependent manner. Regular supplementation in combination with a Mediterranean-style diet, regular exercise, and medical therapy improve CV risk, endothelial function, and vascular reactivity in MetS women, although, to date, very little data are available concerning the effect of genistein on heart function [12,14,15,16,17,18].

The aim of this study was therefore to investigate whether genistein therapy might influence heart function in postmenopausal patients with MetS.

## 2. Materials and Methods

### 2.1. Design and Setting

A group of postmenopausal women, affected by type-2 DM and free from previous CV events, was enrolled from the population of postmenopausal women referred to the Department of Clinical and Experimental Medicine (University Hospital, Messina, Italy) for MetS. All patients were of Caucasian origin. This study was planned as a sub-study aimed to investigate the influence of genistein on heart function, from the original randomized multicenter clinical trial (clinicaltrials.gov registration NCT00541710) managed in collaboration with the University of Palermo (Palermo, Italy) and the University of Magna Graecia (Catanzaro, Italy) [16]. All patients gave written informed consent and the Ethical Committee of the University Hospital of Palermo approved the protocol of this study (approval number: RODA-12254).

Diagnosis of MetS was made considering the presence of at least three criteria among those provided by the modified US National Cholesterol Education Program Adult Treatment Panel III [19], as follows: (a) waist circumference >88 cm; (b) triglycerides >8.3 mmol/L or on drug treatment for elevated triglycerides; (c) high-density lipoprotein cholesterol (HDL-C) <2.8 mmol/L or on drug treatment for reduced HDL-C; (d) type-2 DM, according to current guidelines [20]; (e) blood pressure >130/90 mm Hg or on anti-hypertensive therapy. Criteria for menopause included: absence of menstrual period in the preceding year, follicle-stimulating hormone level >50 IU/L, and 17β-estradiol serum level ≤100 pmol/L (≤27 pg/mL) [2,3].

Family history, physical examination, measurement of body surface area (BSA, m^2^), waist circumference (cm) and body mass index (BMI, kg/m^2^), routine laboratory sampling (fasting glucose, total cholesterol, HDL-C, low-density lipoprotein cholesterol (LDL-C), triglyceride levels), fasting serum insulin, and the insulin resistance index by the homeostasis model assessment for insulin resistance (HOMA-IR) were carried out at baseline, 6 months, and 12 months of therapy. Visfatin, adiponectin, and homocysteine (HCY) serum levels were also evaluated, as previously described [16].

Patients with a previous or current cardiovascular event, renal or hepatic failure, coagulopathy, cancer, use of sex hormones or estrogen receptor modulators, steroids, long term treatment with non-steroidal anti-inflammatory drugs, alcohol abuse, or those who smoked more than two cigarettes per day were excluded from the study.

Hypoglycaemic and/or anti-hypertensive drugs were continued during the study period, if required. Fasting glucose target <8.3 mmol/L and blood pressure ≤135/85 mm Hg were recommended to be achieved in all study participants.

Left ventricular (left ventricle, LV) systolic function was evaluated as the primary endpoint to analyze the effect of genistein on cardiac function. In particular, LV systolic function and left atrial (left atrium, LA) morphofunctional indices were investigated according to high-resolution strain-echocardiography imaging measurements.

### 2.2. Randomization

**Figure 1:**
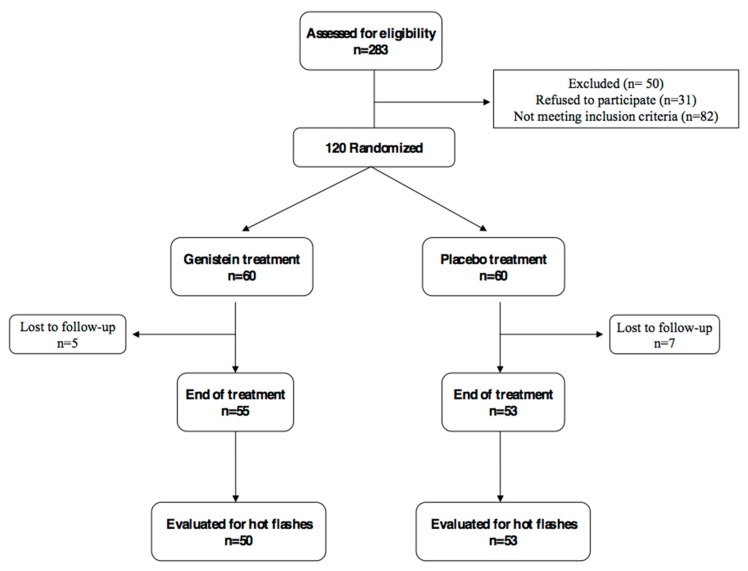
CONSORT diagram (Consolidated Standards of Reporting Trials diagram). The first 11 patients of each arm underwent ultrasound study.

Patients from the original trial were assigned by using a computer–generated double–blind randomization sequence to receive genistein (genistein group) or placebo (control group) twice a day. Both tablets were supplied by Mastelli srl (Sanremo, Italy). The genistein daily dose was 54 mg and tablets were identical to the placebo in appearance and taste. Genistein serum levels were measured both at baseline and the final visit, as previously described [16,21]. Only the first 11 patients of each group underwent ultrasound studies, due to a budget reduction from the original proposal (Figure 1). Patients were enrolled according to the computer–generated double–blind randomization sequence, using the first randomization numbers for each group and maintaining the blinding of researchers directly involved in the study. Genistein and placebo were given for 12 months.

Sample size was calculated in the parent study to provide 80% power to detect an expected absolute between-group difference in HOMA-IR of 20% after one year of treatment, assuming a two-tailed level of 0.05 as significant [16].

### 2.3. Diet and Exercise

Patients were all recommended to follow a Mediterranean-style dietary regimen (25–30% fat, less than 10% saturated fatty acids, 55–60% carbohydrates, and 15% protein), but further intake of soy products or supplements was discouraged.

Regular exercise, such as walking or biking for 80 to 100 min per week, was also suggested to participants and was recorded weekly for each patient. Dietary and treatment adherence was recorded at each visit.

### 2.4. Electrocardiogram and Echocardiogram

After performing a conventional 12-lead electrocardiogram (ECG), ultrasound study was carried out with a commercially available station (Esaote Mylab 30, Florence, Italy), equipped with an M–mode, two–dimensional color–Doppler and strain-feature analysis. Imaging was achieved by the conventional five trans-thoracic views (parasternal long-axis and short-axis, apical 2, 4, and 5 chambers). Quantitative findings were measured as the mean value of three consecutive beats. Simultaneous ECG monitoring was carried out in each patient. Left ventricular diameters, wall thickness, mass, systolic and diastolic volumes, ejection fraction (Simpson’s rule biplane method), LA area and fractional area change [(systolic area minus diastolic area)/systolic area %], as well as LA and LV longitudinal shortening, were calculated according to recommendations of both the American and European Societies of Echocardiography [22]. Cardiac chamber volumes and mass were indexed to body surface area.

Left ventricular systolic function strongly related to ejection fraction. Moreover, LV shape, LV strain analysis, mitral annulus posterior systolic excursion (MAPSE), and longitudinal function (tissue S’ velocity) were also evaluated.

We also investigated LV diastolic function by PW-Doppler, sampling the mitral inflow early and late diastolic velocities (E–wave and A–wave, respectively, and E/A ratio) and tissue–Doppler velocity both at basal septum level and lateral mitral annulus (E’ velocity, A’ velocity and E/E’ ratio).

**Figure 2:**
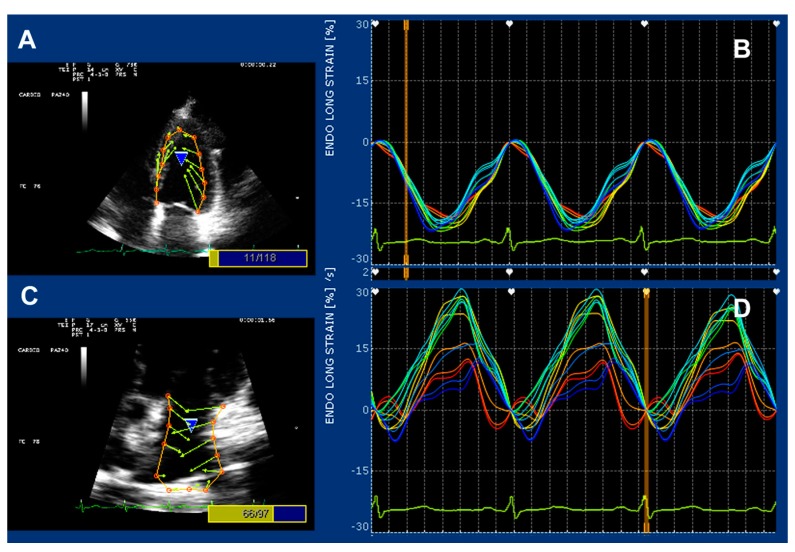
Strain–featured processing from the four–chamber apical view in a genistein patient. Vectors and curves are generated by processing 13 endocardial points on either the left ventricular (panels (**A**) and (**B**)) or the left atrial wall (panels (**C**) and (**D**)).

A dedicated strain software package (X–Strain TM by Esaote, Florence, Italy) was used for both LA and LV longitudinal strain [23]. Good quality imaging with an adequate frame rate (50–70 frames per second) was needed for this purpose. Data were digitally stored and analyzed by a Fourier equation that warrants heart motion periodicity–based accuracy. Strain curves were generated by a feature–tracking mode (Figure 2). Strain measurements were generated by processing the region of interest (several endocardial points from either parasternal short axis or apical 4-chamber view). Left ventricular longitudinal and circumferential strains are negative, whereas radial strain and peak atrial longitudinal strain (PALS) are positive values.

### 2.5. Statistical Analysis

Descriptive statistical analyses were performed to evaluate basal demographic and clinical characteristics. All results were expressed as medians with interquartile range (IQ) for continuous variables, and absolute and percentage frequencies for categorical variables.

All variables were evaluated at basal time and after 12 months of treatment, and percentage changes from baseline were evaluated at the end of the treatment in both genistein and placebo patients to determine the differences between groups.

The Kolmogorov-Smirnov test for normality was performed to evaluate normal distribution. Since some of the numerical variables were not normally distributed and the low sample number did not guarantee valid asymptotic results, a non-parametric approach was used.

The U Mann–Whitney test for independent values was applied to compare characteristics of the randomized subjects, according to treatment group. In addition, differences within group for paired measurements were tested by the Wilcoxon signed-rank test analysis.

Statistical analyses were performed using Statistical Package for Social Science (SPSS Statistics 17.0, Chicago, IL, USA) software for Windows package. *p* < 0.05 was considered statistically significant.

## 3. Results

### 3.1. Baseline Characteristics

A total of 22 patients, aged 55 (IQ 6) years, were enrolled from the main randomized controlled trial: 11 from the genistein and 11 from the control group, respectively. No differences in clinical, laboratory, or morphofunctional values between groups were shown at basal. Adherence to diet and exercise was similar between groups. All patients met the criteria for MetS diagnosis. Eighteen patients (82%), nine in each group, showed mild to moderate systemic hypertension with no differences in systolic-diastolic mean pressure between groups. Waist circumference >88 cm was measured in eight (73%) genistein patients and nine (82%) controls (ns). Five (45%) and six (54%) patients were obese in the genistein and placebo groups, respectively. The characteristics of patients in each group at basal and after 12 months are described in Table 1. Resting ECG was normal in all patients. None of patients were treated with glitazones. Beta blockers users were one in the genistein and two in the placebo group. Angiotensin-converting enzyme inhibitors or Angiotensin II receptor antagonist users were nine in the genistein and 10 in the placebo group.

### 3.2. Clinical and Laboratory Results at Follow-Up

All patients completed the study. Genistein levels increased from 15 nmol/L (95%CI 7.3–22.6) at baseline to 780 nmol/L (95%CI 741.7–818.3) at 12 months in treated subjects. BMI and waist values were similar at the end of follow-up in both groups. Resting blood pressure was on target in the majority of patients (90%) and no differences from basal to the end of follow-up or between groups were observed. Fasting glucose and insulin levels were similar at the beginning and at the end of follow-up in both groups. After 12 months of treatment, HOMA-IR (*p* = 0.007), visfatin (*p* = 0.016), and homocysteine (*p* = 0.034) significantly decreased in the genistein group compared to the controls. Conversely, serum adiponectin increased in the genistein, but not significantly, compared to the control group at the end of the treatment (Table 2). Changes in total cholesterol, LDL cholesterol, HDL cholesterol, and triglycerides at the end of the treatment were similar between groups.

### 3.3. Cardiac Morphofunctional Indices

Adequate ultrasound imaging was attained from each patient. Changes in cardiac morphology and function are described in Table 1 and Table 2. Both LV ejection fraction (*p* = 0.040) and LA fractional area change (*p* = 0.034) significantly increased in the genistein patients compared the to controls during the treatment.

Additionally, LV ejection fraction (*p* = 0.009), tissue systolic wave velocity at lateral mitral annulus (*p* = 0.022), LA volume (*p* = 0.041), LA fractional area change (*p* = 0.035), and PALS (*p* = 0.021) also significantly improved in genistein-treated patients compared to basal, whereas no differences were shown in the control group. Advanced echo-strain analysis showed no relevant changes in LV radial, circumferential, and longitudinal global deformation (Table 2). No advanced LV diastolic dysfunction was observed. Grade 1–2 diastolic dysfunction was present at enrollment in two genistein patients and one control. However, at the end of follow-up, just one patient in the genistein group and four in the placebo group showed diastolic anomalies.

### 3.4. Adverse Events and Side-Effects

No clinically relevant side effects, flushing, or palpitations were reported. Nobody experienced serious arrhythmias or cardiac symptoms.

## 4. Discussion

Recent findings indicate that approximately 25% of the USA population shows traits of MetS, and the constellation of symptoms becomes of clinical concern particularly in postmenopausal patients with DM and hyperlipemia, whose CV risk is higher than that in the general population. Moreover, low adherence to treatment with lipid-lowering drugs is particularly high in female patients, strongly influenced by reimbursement criteria revision, and educational programs in primary care result in challenging efficacy in reaching the clinical targets. [8,9,24,25]. As a consequence, the increasing prevalence of MetS over the last decade hinders the efforts of physicians to improve the CV risk profile through better glycaemic homeostasis [1,2,6,26].

Our group has previously recognized an improvement in the HOMA-IR and several markers of CV risk of MetS postmenopausal patients treated with genistein [14,15,16]. Furthermore, the present findings suggest an interesting impact of genistein treatment on cardiac function in this clinical setting [12].

Using the strain deformation analysis, the most advanced technique to discover early and subclinical LV impairment at the present time [23], our results suggest that one year of treatment with genistein at the dose of 54 mg/day did not worsen LV chamber size or function. On the contrary, genistein treatment improved LV ejection fraction. Such a gain in LV systolic function cannot be exclusively attributed to a direct genistein effect on myocardium, but could rather depend on several factors, including the improvement in adipokine serum levels. In addition, the lack of improvement in the control group emphasizes the efficacy of the treatment with genistein. Similarly, the improvement in the LA size and function has been limited to the genistein group. These patients showed a decrease in LA systolic volume index together with a raise in fractional area change and PALS, without changes in systo-diastolic blood pressure. This finding is particularly interesting since patients with DM and hypertension, or MetS, have an increased risk of atrial fibrillation and heart failure [27,28,29].

Therefore, the present study suggests that genistein could also reduce the LA afterload and potential dysfunction. In fact, hypertensive, diabetic, and overweight patients have an increased risk of atrial dysfunction. Significant changes in LA volume and longitudinal deformation might contribute to the reduction of LA dilatation, fibrosis, and the occurrence of atrial fibrillation, regardless of the loading conditions at the time of examination [27,28].

The cardioprotective effects of genistein could be partially justified by the improvement in the adipokine profile. Adiponectin increased significantly over the 12 months in both groups and the change was not significantly different between groups. Adiponectin promotes insulin-sensitizing effects and peripheral glucose use, as previously reported [16]. Low circulating levels of adiponectin were found in obese patients with type-2 DM, as well as in postmenopausal MetS women [30,31,32,33]. Adiponectin is a modulator of vascular remodeling, and low serum adiponectin levels are predictors of atherosclerosis and myocardial infarction, and are associated with higher incidence of cardiovascular events [34]. These effects are probably related to the anti-inflammatory action of adiponectin, in fact, it reduces the production of inflammatory cytokines and adhesion molecules in endothelial cells [35].

On the contrary, the pro-inflammatory visfatin was reduced by genistein after one year of treatment. Visfatin increase in type-2 DM patients was related to heart failure and acute coronary syndromes [36,37,38,39]; visfatin appears to mediate vascular endothelial inflammation by inducing the expression of adhesion molecules (VCAM-1 and ICAM-1) and pro-inflammatory cytokines such as IL-1β, IL-6, and TNF-α [40]. These effects on adipokines might explain the observed protective effect on myocardium. Genistein, by reducing HOMA-IR, may also decrease glucose–mediated cardiotoxicity, lowering glycated proteins and reactive oxygen species, which are involved in the pathogenesis of interstitial fibrosis in diabetic cardiomyopathy [41,42].

Among the pro-inflammatory factors, homocysteine, which has been suggested as an independent risk factor for endothelial dysfunction and atherosclerosis [43], was also reduced by genistein. Since homocysteine could indirectly impact cardiac function in women with MetS, its reduction in genistein-treated patients confirms our previous data obtained in healthy postmenopausal subjects [16] regarding the possible cardioprotective role of this isoflavone.

Despite the intriguing present results, some limitations of the study should be taken into account. Our results were obtained from a population without signs of cardiac dysfunction and all women were of Caucasian origin, thus we do not know if the same effects could be reproduced in different ethnic groups. The small sample size is a limitation of the present study and no definitive conclusions can be drawn from our proof of concept study. However, this study was able to demonstrate, for the first time, that genistein significantly improved cardiovascular function in postmenopausal women with metabolic syndrome. Accordingly, using this preliminary information, a future larger study expressly designed to evaluate the effect of genistein on cardiac function is encouraged.

In conclusion, our preliminary data suggest that a daily supplementation with 54 mg of pure genistein improves both LA remodeling and LV function in postmenopausal women with MetS.

## Figures and Tables

**Table 1:** Clinical, laboratory, and morphofunctional values measured at baseline and after 12 months, stratified by treatment groups.

Parameters	Genistein			Controls		
	Basal Median (IQ)	12 Month Median (IQ)	# *p* Value	Basal Median (IQ)	12 Month Median (IQ)	# *p* Value
Body mass index (kg/m^2^)	30.3 (5.0)	31.5 (7.0)	0.167	30.4 (11.2)	29.9 (10.8)	0.593
Waist circumference (cm)	105.0 (16.0)	104.0 (25.0)	0.798	99.0 (19.0)	98.0 (17.0)	0.438
Systolic blood pressure (mm Hg)	130.0 (38.0)	120.0 (40.0)	0.075	130.0 (30.0)	130.0 (20.0)	0.320
Diastolic blood pressure (mm Hg)	80.0 (15.0)	70.0 (30.0)	0.085	80.0 (10.0)	80.0 (0.0)	0.496
Fasting glucose (mmol/L)	7.1 (2.7)	7.0 (0.9)	0.099	7.8 (2.2)	6.5 (4.3)	0.959
Total cholesterol (mmol/L)	4.9 (1.4)	4.2 (0.7)	0.003	4.9 (1.1)	4.4 (1.1)	0.051
LDL-cholesterol (mmol/L)	2.9 (1.1)	1.6 (0.8)	0.009	2.8 (1.3)	2.5 (0.6)	0.120
HDL-cholesterol (mmol/L)	1.3 (0.7)	1.5 (0.6)	0.093	1.3 (0.4)	1.4 (0.6)	0.798
Triglycerides (mmol/L)	1.5 (0.9)	1.2 (0.8)	0.008	1.4 (0.8)	1.2 (0.7)	0.075
Insulin levels (nUI/L)	9.7 (7.8)	8.5 (5.3)	0.050	12.2 (12.3)	13.4 (17.9)	0.477
HOMA-IR	3.4 (1.5)	3.0 (1.7)	0.010	3.7 (4.8)	4.7 (5.2)	0.110
Visfatin (ng/mL)	3.1 (2.0)	1.3 (0.7)	0.016	2.1 (2.2)	2.4 (1.3)	0.477
Adiponectin (μg/mL)	6.2 (1.1)	8.0 (5.9)	0.003	5.3 (2.6)	6.2 (2.6)	0.021
Homocysteine (μmol/L)	13.3 (15.3)	10.0 (2.2)	0.003	17.5 (10.8)	14.2 (7.7)	0.091
***Left Ventricle***						
End-diastolic diameter (mm)	47.4 (9.2)	46.2 (8.2)	0.929	45.8 (7.7)	44.2 (5.1)	0.859
BSA-index mass (g/m^2^)	82.9 (33.7)	73.0 (27.8)	0.657	78.6 (15.3)	84.7 (19.9)	1.000
Height-indexed mass (g/m^2.7^)	38.0 (27.7)	44.6 (16.9)	0.328	41.1 (7.9)	39.2 (11.6)	0.859
End-diastolic volume (mL)	67.0 (14.9)	65.0 (26.0)	0.533	72.0 (23.0)	70.0 (28.0)	0.894
BSA-index end-diastolic vol. (mL/m^2^)	40.3 (13.9)	37.9 (10.5)	0.328	37.2 (10.9)	39.2 (15.1)	0.790
End-diastolic shape	0.6 (0.09)	0.6 (0.1)	0.241	0.6 (0.08)	0.6 (0.1)	0.859
Ejection fraction	0.6 (0.04)	0.7 (0.05)	0.009	0.7 (0.11)	0.7 (0.10)	0.790
MAPSE (mm)	15.8 (4.1)	16.5 (1.3)	0.182	16.3 (4.8)	16.0 (4.9)	0.790
TDV Septal S’-wave (cm/s)	10.0 (3.0)	10.0 (2.0)	0.764	8.0 (4.0)	8.0 (3.0)	0.258
TDV Lateral S’-wave (cm/s)	10.0 (1.0)	12.0 (2.0)	0.022	10.0 (2.0)	11.0 (3.0)	0.305
***Left Ventricular Diastolic Indices***						
Mitral E/A velocity ratio	1.0 (0.23)	0.8 (0.33)	0.449	0.8 (0.44)	0.9 (0.28)	0.965
Mitral E-wave DT (ms)	204.0 (16.0)	216.0 (13.0)	0.075	223.0 (24.0)	242.0 (27.0)	0.059
E/E’ velocity ratio	6.8 (1.0)	7.5 (2.6)	0.533	7.2 (3.5)	7.8 (3.3)	0.859
***Left Ventricular Global Strain Measurements***						
Radial Strain (%)	32.0 (10.9)	30.0 (15.0)	0.789	36.0 (9.5)	27.9 (12.3)	0.110
Circumferential Strain (%)	−21.0 (7.7)	−22.5 (4.9)	1.000	−23.5 (9.2)	−25.3 (5.0)	0.594
Longitudinal Strain (%)	−17.5 (1.5)	−17.3 (1.6)	0.328	−15.8 (4.7)	−18.8 (4.9)	0.213
***Left Atrium***						
BSA-indexed ES volume (mL/m^2^)	29.5 (4.2)	22.6 (11.0)	0.041	32.3 (10.2)	27.5 (7.2)	0.248
Fractional area change (%)	37.0 (6.0)	41.0 (6.0)	0.035	35.0 (2.0)	35.0 (5.0)	0.510
PALS (%)	25.5 (5.8)	31.0 (8.5)	0.021	23.6 (10.2)	24.0 (6.5)	0.929

Values are expressed as medians with interquartile range (IQ); BSA, body surface area; DT, deceleration time; E/A, early/late diastolic velocity through the mitral inflow; E/E’ velocity ratio, ratio between mitral E velocity and tissue E velocity; ES, end-systolic; HDL high-density lipoprotein; HOMA-IR, homeostasis model assessment for insulin resistance; LDL, low-density lipoprotein; MAPSE, mitral annular posterior systolic excursion; PALS, peak atrial longitudinal strain; S’, tissue systolic velocity; TDV, tissue Doppler velocity; # *p*-values were calculated using the Wilcoxon rank test for each group, from baseline to the end of the treatment (12 months) as well as within-group comparisons.

**Table 2:** Variations of clinical, laboratory values, and morphofunctional findings at Doppler echocardiography after 12 months of treatment: between groups comparison.

Parameters	Percentage Changes from Baseline after 12 Months Treatment		
	Genistein Median (IQ)	Controls Median (IQ)	# *p* Value
Body mass index (kg/m^2^)	1.6 (5.7)	0.9 (4.5)	0.171
Waist circumference (cm)	0.0 (4.9)	0.0 (4.4)	0.898
Systolic blood pressure (mm Hg)	0.0 (11.8)	0.0 (12.2)	0.076
Diastolic blood pressure (mm Hg)	−8.3 (12.5)	0.0 (6.7)	0.057
Fasting glucose (mmol/L)	−7.1 (23.5)	−0.9 (18.3)	0.332
Total cholesterol (mmol/L)	−12.4 (14.9)	−15.5 (23.3)	0.748
LDL-cholesterol (mmol/L)	−18.1 (45.1)	−16.8 (43.2)	0.270
HDL-cholesterol (mmol/L)	16.3 (27.6)	−2.1 (15.6)	0.243
Triglycerides (mmol/L)	−12.5 (13.8)	−21.5 (56.6)	0.898
Insulin levels (nUI/L)	−21.5 (27.9)	1.7 (81.0)	0.193
HOMA-IR	−19.7 (39.0)	18.0 (71.2)	0.007
Visfatin (ng/mL)	−50.9 (73.2)	−7.0 (32.2)	0.016
Adiponectin (μg/mL)	29.4 (62.8)	12.6 (38.6)	0.088
Homocysteine (μmol/L)	−24.3 (34.4)	−8.1 (50.1)	0.034
***Left Ventricle***			
End-diastolic diameter (mm)	3.2 (14.7)	0.2 (26.5)	0.949
BSA-index mass (g/m^2^)	2.9 (30.4)	10.5 (41.6)	0.748
Height-indexed mass (g/m^2.7^)	16.3 (22.2)	−5.4 (45.1)	0.519
End-diastolic volume (mL)	−1.3 (45.5)	−7.8 (67.8)	0.898
BSA-index end-diastolic vol. (mL/m^2^)	−0.4 (49.1)	7.0 (48.5)	1.000
End-diastolic shape	3.2 (5.8)	−1.6 (7.6)	0.606
Ejection fraction	20.3 (12.5)	−1.7 (24.8)	0.040
MAPSE (mm)	4.4 (38.0)	0.6 (26.4)	0.606
TDV Septal S’-wave (cm/s)	0.0 (26.5)	−12.5 (48.1)	0.332
TDV Lateral S’-wave (cm/s)	10.0 (9.1)	9.1 (31.3)	0.606
***Left Ventricular Diastolic Indices***			
Mitral E/A velocity ratio	−9.8 (34.8)	6.0 (56.8)	0.652
Mitral E-wave DT (ms)	10.0 (12.4)	6.0 (11.9)	0.748
E/E’ velocity ratio	6.4 (72.6)	3.9 (49.9)	0.748
***Left Ventricular Global Strain Measurements***			
Radial Strain (%)	12.6 (44.3)	−13.4 (35.1)	0.133
Circumferential Strain (%)	0.9 (30.9)	3.7 (38.9)	0.797
Longitudinal Strain (%)	−2.8 (11.3)	4.3 (24.9)	0.243
***Left Atrium***			
BSA-indexed ES volume (mL/m^2^)	−22.6 (47.7)	−20.8 (61.1)	0.151
Fractional area change (%)	11.1 (22.6)	2.8 (9.5)	0.034
PALS (%)	13.3 (25.4)	−11.2 (51.3)	0.270

Values are expressed as medians with interquartile range (IQ); BSA, body surface area; DT, deceleration time; E/A, early/late diastolic velocity through the mitral inflow; E/E’ velocity ratio, ratio between mitral E velocity and tissue E velocity; ES, end-systolic; HDL high-density lipoprotein; HOMA-IR, homeostasis model assessment for insulin resistance; LDL, low-density lipoprotein; MAPSE, mitral annular posterior systolic excursion; PALS, peak atrial longitudinal strain; S’, tissue systolic velocity; TDV, tissue Doppler velocity; # *p*-values were calculated using the Wilcoxon rank test for each group, from baseline to the end of the treatment (12 months) as well as within-group comparisons.
